# Meat

**Published:** 1871-07

**Authors:** 


### MEAT.

Meat is a wholesome and strengthening food, when properly selected, killed and cooked.—Meat supplies us with the necessary material for the manufacture of muscle, and with most of the salts necessary to nutrition.

The process of preparing meat for the market, as usually practiced, is wrong. Too much of the blood is removed in slaughtering. Nearly one-fourth of the ox is solid flesh, and about one-fifth blood. It is found that blood and flesh are identical in chemical composition, and their nutritive value are equal; blood, by reason of its fluid condition, is more readily digested, therefore, most valuable for the purposes of nutrition. In removing it from the carcass by too free bleeding, the phosphates, sulphates, iron, and potash, escape from the flesh with the last flowing of the blood, thereby greatly diminishing the nutritive materials intended to be furnished. Meat from which the blood is almost completely extracted, is acid and unwholesome. Game and fish, being but slightly deprived of blood, are more easily digested, and owe their distinctive tastes to this fact. The extreme of muscular vigor is attained by eating meat that has not been too much deprived of its fluid flesh.

Salt meat is objectionable for the following reasons:—Its soluble salts are extracted with the brine, and as it must be steeped in water before being used, much of the soluble, nutritive matter is afterwards dissolved and lost in the water, while the covering of the fibres of the meat is so hardened that it requires a long time to undergo digestion.

In preparing animal food for use, the following facts should be observed:—Boiling lessens the weight of meat nearly one-third. To retain the nutritive principles of the meat, therefore, the piece should be large, *and should be plunged into boiling water*, instead of placing it in a pot of cold water and so left to come to the boiling point. By placing it into the boiling water, the outer crust of the meat is hardened quickly, which in a great measure, retains the juice within. In making soup or broth the rule should be reversed as it is then desirable to extract the juice. In roasting, the loss of weight occurs mainly in water and fat, the juice being retained by the crust the fire soon produces on the outside; roasted meat is therefore more nutritious than that which has been boiled. Rare, roast beef is the most nutritious animal food that is furnished us in our markets. Venison, and other wild meats are sooner digested, but are not so rich in nutritive qualities. If you desire all the nutrition possible to be derived from meat, let your selection be beef, roasted in a hot oven, and served rare.

